# Global estimates of service coverage for severe mental disorders: findings from the WHO Mental Health Atlas 2017

**DOI:** 10.1017/gmh.2021.19

**Published:** 2021-07-21

**Authors:** Kara Jaeschke, Fahmy Hanna, Suhailah Ali, Neerja Chowdhary, Tarun Dua, Fiona Charlson

**Affiliations:** 1 Policy and Evaluation Group, Queensland Centre for Mental Health Research, Wolston Park Rd, Wacol QLD 4076, Australia; 2 School of Population Health, University of Queensland, St Lucia QLD 4072, Australia; 3 Department of Mental Health and Substance Abuse, World Health Organization, Geneva, Switzerland; 4 Institute for Health Metrics and Evaluation, University of Washington, Seattle, WA 98195, USA

**Keywords:** Universal health coverage, mental disorders, psychosis

## Abstract

**Background:**

The study estimated service coverage for severe mental disorders (psychosis, bipolar disorder and moderate-severe depression), globally and regionally, using data collected from the Mental Health Atlas 2017.

**Methods:**

Service coverage was defined as the proportion of people with a disorder contacting a mental health service among those estimated to have the disorder during a 12-month period. We drew upon 12-month service utilisation data from the Mental Health Atlas 2017. Expected prevalent cases of individual disorders were estimated using the disorder-specific prevalence rate estimates of the Global Burden of Disease Study 2016 and total population sizes. Methods for assessing the validity of country-reported service utilisation data were developed and applied.

**Outcomes:**

From 177 countries, 50 countries provided reliable service coverage estimates for psychosis, along with 56 countries for bipolar disorder, and 65 countries for depression. The mean service coverage for psychosis was lowest in low- [10.9% (95% confidence interval (CI) 3.3–30.4)] and lower middle-income countries [21.5% (95% CI 11.9–35.7)] and highest in high-income countries [59.5% (95% CI 42.9–74.1)]. Service coverage for bipolar disorder ranged between 3.1% (95% CI 0.8–11.5) and 10.4% (95% CI 6.7–15.8). Mean service coverage for moderate-severe depression ranged between 2.9% (95% CI 1.3–6.3) for low-income countries and 31.1% (95% CI 18.3–47.6) for high-income countries.

**Interpretation:**

The reporting method utilised by the Mental Health Atlas appears to be reliable for psychosis but not for depression. This method of estimating service coverage provides progress in tracking an important indicator for mental health; however, it highlights that considerable work is needed to further develop global mental health information systems.

## Introduction

Mental disorders contribute a substantial burden globally, in terms of both morbidity and mortality, and significant social and economic impacts (Whiteford *et al*., 2015). However, despite the availability of evidence-based interventions, the prevalence of mental disorders is not reducing over time (Jorm *et al*., 2017). This finding highlights the need to deliver quality treatment. Yet, the available data suggest that, globally, a significant proportion of people with mental disorders receive no treatment, disproportionately so in low- and middle-income countries (LMICs) (Demyttenaere *et al*., 2004).

In 2013, the World Health Assembly adopted the World Health Organization's (WHO) Comprehensive Mental Health Action Plan 2013–2020, which articulated practical guidelines for addressing mental health, especially in LMICs (World Health Organization, 2013). A key objective of the Action Plan was to strengthen mental health information systems. In 2015, the United Nations General Assembly adopted a new global development agenda, comprising of 17 integrated and indivisible Sustainable Development Goals (SDGs) (UN General Assembly, 2015). Mental health is directly referred to in SDG Goal 3 and is relevant across numerous others (Lund *et al*., 2018) – requiring the international community to track and monitor mental health indicators.

One indicator that has emerged with considerable utility for mental health is ‘service coverage’, specifically ‘contact coverage’ (Tanahashi, 1978). Service coverage for mental health is a key target in the WHO Mental Health Action Plan; however, methodological difficulties are associated with producing robust estimates of service coverage, and existing attempts to do so on a global scale have been limited. The World Mental Health Survey Initiative (WMHS) conducts national face-to-face surveys to obtain accurate cross-national information about the prevalence, correlates and service utilisation of mental and substance use disorders. At the time of writing, the WMHS have been conducted in 29 countries, representing all regions of the world (Harvard University, 2005). While population surveys are a robust method for estimating service coverage, they are resource-intensive and not a feasible method for repeated measurement of the service coverage indicator.

The WHO Assessment Instrument for Mental Health Systems (WHO-AIMS) was one of the most comprehensive WHO tools for collecting information on the mental health systems in LMICs and enabled analysis of the availability and accessibility of services in more than 80 countries (World Health Organization, 2005; Lora *et al*., 2012). The WHO-AIMS exercise highlighted how global service coverage data are fragmented, frequently limited in its scope, and is not standardised across health systems to facilitate comparisons and monitoring of changes over time, even in high-income countries with well-developed mental health systems. While WHO-AIMS represented a valuable tool providing an in-depth view of the current status of mental health systems, another tool was designed by WHO for more routine use – the Mental Health Atlas.

Since 2001, WHO's Mental Health Atlas has established itself as the most comprehensive and widely used source of information on countries' mental health activities. Starting with Mental Health Atlas 2011, the Atlas series assumed new importance. It provides a tool for monitoring Member State mental health systems and is the most comprehensive source of information relating to mental health on a global scale (World Health Organization, 2014, 2018). It also represents a critical component of tracking the progress of service coverage as an important mental health indicator. The Mental Health Atlas collects data across five domains: (1) global reporting on core mental health indicators; (2) mental health system governance; (3) financial and human resources for mental health; (4) mental health service availability and uptake; (5) mental health promotion and prevention.

Within the WHO Member States, the mental health focal points in the Ministries of Health of each country were the main Atlas project collaborators. In consultation with other countries' experts, these focal points provided answers to the Mental Health Atlas 2017 questionnaire. During the countries' questionnaire submission, the WHO supported and provided guidance to the focal points. The WHO screened the completed questionnaires for inconsistencies and contacted respondents to ensure the quality of data.

It has been noted that although the Mental Health Atlas 2011 and 2014 represent the most accurate and complete information on national mental health service provision available, the data have several limitations. These include poor quality, missing data and insufficient standardisation of country reports and definitions. The Mental Health Atlas 2017 was revised to address some of these limitations. This study aims to estimate service coverage for severe mental disorders (psychosis, bipolar disorder and moderate-severe depression), globally and regionally, using data collected from the Mental Health Atlas 2017; while simultaneously assessing whether the feasibility and appropriateness of using these data for estimating service coverage varies between individual mental disorders.

## Methodology

This study adheres to the Guidelines of Acute and Transparent Health Estimates Reporting (GATHER) Statement (Stevens *et al*., 2016). The GATHER checklist can be found in the online Appendix Table S1.

**Fig. 1.**
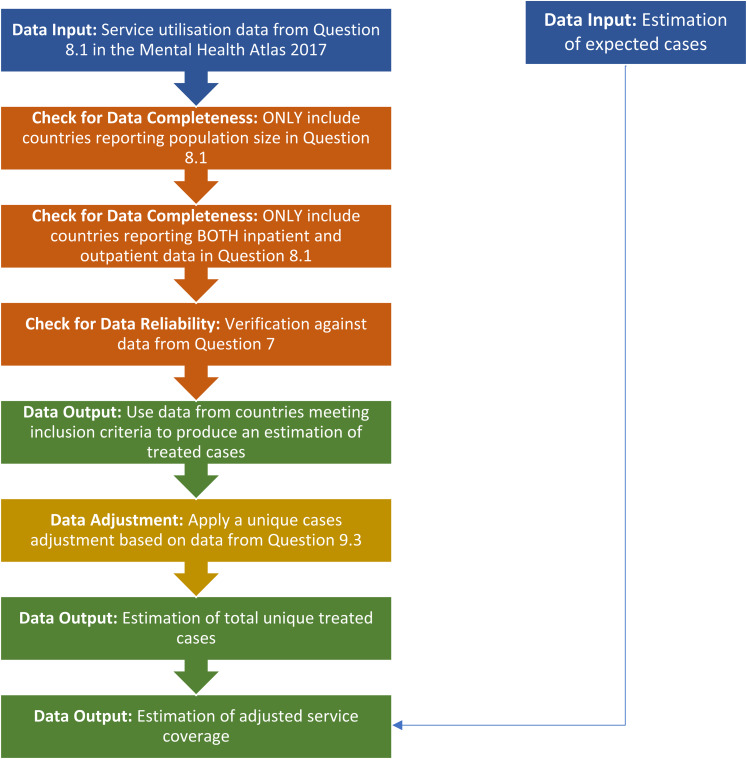
Flow of data inputs, adjustments and outputs.

This study uses data from the Mental Health Atlas 2017. The 2017 edition of Mental Health Atlas uses 2016 data. In order to include data from individual countries in the final service coverage estimates, data were first required to undergo a process (Fig. 1). This comprised identifying data inputs, determining if data were complete and reliable, adjusting data as required and comparing data outputs. Data were reliable if it was consistent between the expected and actual service coverage estimates.

### Service coverage

Service coverage was defined as the proportion of people with a disorder contacting a mental health service (from service utilisation data) among those estimated to have the disorder (population prevalence) during a 12-month period. This specific measurement has been termed ‘contact coverage’ by Tanahashi *et al*. and reflects the actual contact between the service provider and the user (Tanahashi, 1978). Unless otherwise specified, we use the more familiar term ‘service coverage’ to represent 12-month contact coverage throughout the remainder of this paper.
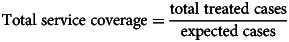


### Treated cases

We drew upon 12-month service utilisation data from the Mental Health Atlas 2017; specifically, data from specialist (inpatient and outpatient) mental health facilities (not primary healthcare) for psychosis, bipolar disorder and depression. Data, including the number of people receiving care from inpatient and outpatient services, population representativeness and population size, were extracted from section 8 of the Mental Health Atlas Questionnaire 2017 into Microsoft Excel 2016 for analysis (online Supplementary Fig. S1). Data were excluded if they did not report on service utilisation of both inpatient and outpatient facilities for a specific disorder, or if countries did not report representativeness of the population that the data were drawn from.

Data from section 7 of the Mental Health Atlas Questionnaire 2017, relating to the number of inpatient and outpatient visits, were used as a validity check of the service utilisation data (online Supplementary Fig. S2). If the number of visits (from section 7) per individual case (from section 8) was less than 1, data were excluded from further analysis.

Total treated cases were calculated according to the following formulas:
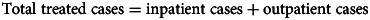

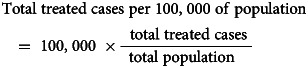


### Expected cases

Expected prevalent cases of individual disorders were estimated using the disorder-specific prevalence rate estimates of the Global Burden of Disease Study (GBD) 2016 (Vos *et al*., 2017) and total population sizes. GBD 2016 prevalence estimates were derived by first conducting systematic reviews of the literature to identify studies reporting the prevalence, incidence, remission and/or excess mortality for individual disorders. These estimates were entered in DisMod-MR 2.1 for analysis, a Bayesian meta-regression tool (Flaxman *et al*., 2015). If no raw epidemiological data were available for a particular location, data from surrounding locations were used to estimate prevalence. Within DisMod-MR 2.1, regions and super-regions were defined according to GBD 2016's classification of broad geographic regions or continents. Additional information on DisMod-MR 2.1 can be accessed elsewhere (Vos *et al*., 2017).
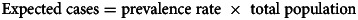


Disorder-specific prevalence rates (point prevalence for depression and schizophrenia, and 12-month prevalence for bipolar disorder) as estimated by GBD 2016 were adjusted in two ways to ensure consistency with service utilisation data for each country that provided Mental Health Atlas 2017 data. Firstly, point prevalence rates of depression were adjusted to 12-month prevalence rates using the inverse of a covariate coefficient which adjusted studies reporting 12-month prevalence to point prevalence. Given the understanding that mild cases of depression are rarely treated in specialist services, we further adjusted depression prevalence estimates to reflect the proportion of cases which are moderate-severe by applying the GBD severity splits [moderate proportion = 0.17, 95% confidence interval (CI) 0.13–0.22; severe proportion = 0.1, 95% CI 0.03–0.2] (Vos *et al*., 2017).

Secondly, schizophrenia prevalence rates were adjusted to non-affective psychoses prevalence rates based on a 0.49 ratio derived from the literature (Kendler *et al*., 1996; Widerlöv *et al*., 1997; Cho *et al*., 2007; Perala *et al*., 2007; Perala *et al*., 2008; Phillips *et al*., 2009; Chang *et al*., 2011; Binbay *et al*., 2012; Jorgensen *et al*., 2014; Moreno-Kustner *et al*., 2016; Chang *et al*., 2017). Non-affective psychoses point prevalence rates were not adjusted to 12-month prevalence rates as no statistically significant difference was observed using a GBD modelling covariate.

If a country did not report its population size or its population size was dramatically different from United Nations (UN) population estimates, UN population estimates were used (Global Burden of Disease Collaborative Network, 2018).

### Case definitions

GBD prevalence estimates and Mental Health Atlas service utilisation data adhere to ICD-10 case-definitions for major depression, schizophrenia and non-affective psychosis, and bipolar disorder. GBD prevalence estimates adhere to the DSM-IV and ICD-10 case definitions for major depression (ICD-10: F32.0–9, F33.0–9), schizophrenia (ICD-10: F20; adjusted to non-affective psychosis as per below) and bipolar disorder (ICD-10: F31.0–F31.6, F31.8–F31.9, F34.0–F34.1).

The Atlas questionnaire requests that service coverage data are reported for non-affective psychosis (ICD-10: F20–29), bipolar disorder (ICD-10: F30–31) and depression (ICD-10: F32.1-3–F33.1-3). For this reason, we have avoided using the diagnostic term ‘major depression’ and instead used ‘depression’ with the understanding that some depression cases may not reach diagnostic thresholds. We also refer to the term ‘psychosis’ for the remainder of the manuscript as representing ‘non-affective psychosis’.

### Service utilisation data adjustments

To prevent double-counting of individuals who had used both inpatient and outpatient facilities, total service coverage rates were adjusted to appropriately reflect the total number of unique cases of individuals utilising mental health facilities. Countries were categorised into inpatient-prioritised countries (i.e. most of the population are treated in inpatient facilities) or outpatient-prioritised countries (i.e. most of the population are treated in outpatient facilities) based on reported data. In inpatient-prioritised countries, the data were not adjusted. In outpatient-prioritised countries, it was assumed that all individuals utilising inpatient facilities also utilised outpatient facilities.

The total unique cases adjustment was based on question 9.3 of the Mental Health Atlas 2017 questionnaire, which provides a follow-up rate ranging from 1 to 4, as follows:

1. 25% or less of discharged inpatients received a follow-up outpatient visit within one month
2. 26–50% of discharged inpatients received a follow-up outpatient visit within one month
3. 51–75% of discharged inpatients received a follow-up outpatient visit within one month
4. More than 75% of discharged inpatients received a follow-up outpatient visit within one month

The adjusted outpatient estimate was calculated by averaging the follow-up range, multiplying this by the inpatient cases, and subtracting this product from the reported outpatient utilisation value (see formula below). For countries that did not report a follow-up rate, the median follow-up rate for the respective GBD world region was used (Institute for Health Metrics and Evaluation, 2019a). If the adjusted outpatient utilisation value was negative, the original reported outpatient utilisation value was used.

Adjusted service coverage was calculated as follows:
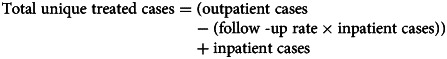

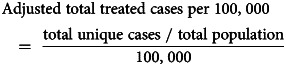

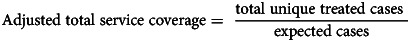


### Service coverage thresholds

Although Question 8 of the Mental Health Atlas requests data on number of cases, health information systems frequently only record number of patient visits. With this knowledge, we designed validity check to determine whether countries reported cases or visits by applying lower and upper service coverage thresholds derived from the published literature (Table 1). For psychosis, the lower threshold was based on WMHS data showing 12-month service use split by severity of mental disorder. For the lower threshold, we took the WMHS country with the lowest service coverage estimate for severe disorders and applied the lower confidence interval of this estimate (Wang *et al*., 2007). This was done for each income group. For depression, the upper threshold was chosen using the same process – by applying the upper confidence interval of the highest service coverage estimate for severe disorders, by income group. For bipolar disorder, the upper limit was also based on WMHS data, which specifically examined 12-month treatment rates for bipolar spectrum disorders (encompassing type I, II and subthreshold bipolar disorder) split by income group (Merikangas *et al*., 2011). Service coverage estimates were excluded if they fell outside of these imposed thresholds.

**Table 1.** Applied lower and upper service coverage estimate thresholds

Income level	Country	Coverage (%)	Lower CI (%)	Upper CI (%)
Psychosis^a^	^b^			
Low income	Nigeria	21.3	1.3	–
Low-middle income	China	11.0	0.4	–
Upper-middle income	Lebanon	20.1	9.9	–
High-income	Japan	24.2	14.4	–
Depression^a^	^c^			
Low income	Nigeria	21.3	–	41.3
Low-middle income	Colombia	27.8	–	37.2
Upper-middle income	Mexico	25.8	–	34.2
High-income	Belgium	60.9	–	78.7
Bipolar disorder^d^	^e^			
Low and low-middle income	All	13.0	–	21.6
Upper-middle income	All	15.9	–	21.8
High-income income	All	28.4	–	32.3

*Note*: missing values mean no thresholds were set.

a Taken from ‘severe’ category of Table 3: 12-month service use by severity of mental disorders in the WMH surveys (29)

b The country listed has the lowest coverage in the WMH surveys for that income group. This is used as the lower threshold for psychosis coverage estimates.

c The country listed has the highest coverage in the WMH surveys for that income group. This is used as the highest threshold for depression coverage estimates.

d Taken from 12-month treatment from ‘any mental health service’ of Table 6: Lifetime and 12-month treatment of DSM-IV/CIDI Bipolar Spectrum Disorders (30).

e For bipolar disorder, there are only global values from the WMH surveys, which are used as the highest threshold for bipolar coverage estimates.

No upper threshold was set for psychosis with the acknowledgement that there is potential for service coverage in specialist mental health services to be high. Conversely, for bipolar disorder and depression, no lower limits were set in order to capture the low rates of service coverage expected for these disorders.

### Calculation of uncertainty and meta-analysis

We calculated the standard error (s.e.) around each countries' service coverage estimates using the following steps:

– Estimate the s.e. of the treated prevalence 
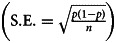
– Estimate the s.e. of the GBD prevalence 
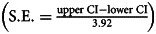
– Estimate the proportion of cases that are treated 
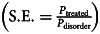
– Estimate the s.e. of the service coverage estimate 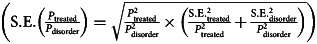

Country-level data were aggregated by World Bank income group (The World Bank, 2018), WHO region (World Health Organization, 2019) and GBD super region (Institute for Health Metrics and Evaluation, 2019b). Random-effects meta-analyses were performed using the metafor package in R (Viechtbauer, 2010). Data were logit transformed prior to analysis using the delta method. Baujat plots were inspected for outliers. Exclusion of influential estimates did not change the final results of the meta-analysis, so they were retained in the final model.

## Results

Following initial exclusions from 177 countries (those not reporting on both inpatient and outpatient facilities or representativeness of population), the sample comprised 70 countries reporting on psychosis, 64 countries reporting on bipolar disorder, and 66 countries reporting on depression. After the WMHS service coverage thresholds were applied, the final numbers were 50, 56 and 65 countries for each disorder, respectively, reporting seemingly reliable service coverage estimates from the Atlas 2017 data. All six WHO regions and all four World Bank income regions were represented.

### Psychosis

Using our methods, seven low- and 14 lower-middle-income countries were found to have reported reliable service utilisation data; providing a mean service coverage of 10.9% (95% CI 3.3–30.4) and 21.5% (95% CI 11.9–35.7), respectively. There were 14 upper-middle-income countries with a mean service coverage of 29.2% (95% CI 19.9–40.7). Fifteen high-income countries reported a mean service coverage of 59.5% (95% CI 42.9–74.1) (Table 2).

**Table 2.** Estimates of service coverage for psychosis using Atlas 2017

Grouping	*n*	Mean (%)	Lower 95% CI	Upper 95% CI
Income				
Low income	7	10.9	3.3	30.4
Lower middle-income	14	21.5	11.9	35.7
Upper middle-income	14	29.2	19.9	40.7
High income	15	59.5	42.9%	74.1
WHO region				
African Region	8	12.8	5.7	26.2
Region of The Americas	11	25.6	14.5	41.2
Eastern Mediterranean Region	6	16.1	3.4	51.4
European Region	16	56.8	44.3	68.4
South-East Asia Region	2	18.4	1.8	73.5
Western Pacific Region	7	34.7	20.2	52.8
GBD region				
High income	11	51.5	35.0	67.6
Latin America and Caribbean	10	26.7	14.1	44.7
Central Europe, Eastern Europe and Central Asia	9	56.5	37.4	73.9
North Africa and Middle East	5	24.3	5.9	62.2
South Asia	2	18.4	1.8	73.5
Sub-Saharan Africa	9	10.0	4.1	22.3
Southeast Asia, East Asia and Oceania	4	25.9	20.3	32.3
Overall	50	31.3	22.9	41.0

Three world regions reported mean service coverage rates above 50% (GBD high-income, GBD Central Europe, Eastern Europe, and Central Asia, and WHO European region). The mean service coverage across all countries was 31.3% (95% CI 22.9–41.0).

### Bipolar disorder

Seven low- and 15 lower-middle-income countries were found to have reported reliable service utilisation data, providing a mean service coverage for bipolar disorder of 3.1% (95% CI 0.8–11.5) and 3.5% (95% CI 1.8–6.8), respectively, per group (Table 3). There were 20 upper-middle-income countries with a mean service coverage of 3.1% (95% CI 1.8–5.5). High-income countries (14 countries) reported the highest mean service coverage at 10.4% (95% CI 6.7–15.9) (Table 3).

**Table 3.** Estimates of service coverage for bipolar disorder using Atlas 2017

Grouping	*n*	Mean (%)	Lower 95% CI	Upper 95% CI
Income				
Low income	7	3.1	0.8	11.5
Lower middle-income	15	3.5	1.8	6.8
Upper middle-income	20	3.1	1.8	5.5
High income	14	10.4	6.7	15.9
WHO region				
African Region	9	2.6	0.9	7.2
Region of The Americas	14	4.2	2.6	6.7
Eastern Mediterranean Region	8	1.4	0.4	5.1
European Region	16	7.2	4.2	12.2
South-East Asia Region	1	21.7	–	–
Western Pacific Region	8	7.5	4.1	13.1
GBD region				
High income	8	14.5	10.3	20.1
Latin America and Caribbean	13	4.1	2.4	6.7
Central Europe, Eastern Europe and Central Asia	11	4.3	2.3	8.0
North Africa and Middle East	7	2.0	0.6	6.9
South Asia	7	2.0	0.6	6.9
Sub-Saharan Africa	10	1.9	0.6	5.6
Southeast Asia, East Asia and Oceania	7	8.5	4.3	16.0
Overall	56	4.4	3.1	6.3

The WHO South-East Asia region reported the highest service coverage for bipolar disorder at 21.7%, although this was based on only one country. This was followed by the GBD high-income region at 14.5% (95% CI 10.3–20.1). The mean service coverage for bipolar disorder across all countries was 4.4% (95% CI 3.1–6.2).

### Depression

The estimated mean service coverage for moderate-severe depression was extremely low overall at 10.7% (95% CI 7.4–15.2) and ranged between 2.9% (95% CI 1.3–6.3) for low-income countries and 31.1% (95% CI 18.3–47.6) for high-income countries (Table 4).

**Table 4.** Estimates of service coverage for moderate to severe depression using Atlas 2017

Grouping	*n*	Mean (%)	Lower 95% CI	Upper 95% CI
Income				
Low income	8	2.9	1.3	6.3
Lower middle-income	16	4.3	2.3	7.9
Upper middle-income	22	13.0	8.0	20.3
High income	17	31.1	18.3	47.6
WHO region				
African Region	10	3.4	2.2	5.3
Region of The Americas	14	14.6	8.2	24.6
Eastern Mediterranean Region	9	3.6	1.5	8.3
European Region	18	30.9	18.9	46.1
South-East Asia Region	3	11.0	1.0	61.1
Western Pacific Region	9	6.1	2.2	15.7
GBD region				
High income	11	35.5	20.1	54.6
Latin America and Caribbean	13	15.0	8.1	26.2
Central Europe, Eastern Europe and Central Asia	10	20.3	8.7	40.3
North Africa and Middle East	8	5.0	2.8	9.0
South Asia	2	3.7	0.6	19.4
Sub-Saharan Africa	11	2.7	1.4	5.0
Southeast Asia, East Asia and Oceania	8	6.9	2.2	19.8
Overall	63	10.7	7.4	15.2

Service coverage for moderate-severe depression was lowest in the WHO African region at 3.4% (95% CI 2.2–5.3), the WHO Eastern Mediterranean region at 3.6% (95% CI 1.5–8.3), the GBD Sub-Saharan Africa region at 2.7% (95% CI 1.4–5.0) and the GBD South Asia region at 3.7% (95% CI 0.6–19.4). Service coverage for depression was highest in the WHO European region at 30.9% (95% CI 18.9–46.1) and the GBD High income region at 35.5% (95% CI 20.1–54.6).

## Discussion

Findings presented in this study represent the largest-scale effort to use service utilisation data from a group of countries, representing all world regions and income groups, to report on the status of service coverage for severe mental disorders. We include 70 countries, compared with 50 countries in a previous analysis by Lora *et al*. using WHO-AIMS data (Lora *et al*., 2012) and 29 countries in which WMHS have been conducted (Harvard University., 2005).

Service coverage estimates for psychosis were the highest across the disorders included in this study. This was also reflected in the only other study providing global service coverage estimates for psychosis (Lora *et al*., 2012). This is likely explained by the fact that psychosis is the most disabling mental disorder that typically requires treatment in specialised mental health services, the services that the Mental Health Atlas focuses on its data collection. The fact that inpatient services typically have the most complete data and contribute significantly to the Mental Health Atlas reporting is another potential explanation for higher service coverage for this condition compared to depression and bipolar.

Our estimates of service coverage for psychosis were similar overall compared to those produced from a previous study using WHO-AIMS data (31% Atlas 2017 *v.* 31% WHO-AIMS). However, there were key differences between the studies. Firstly, this earlier analysis examined schizophrenia, not all psychosis. Additionally, the WHO-AIMS sample of countries included a smaller sample from upper-middle-income countries, and no high-income countries.

This study's 12-month service coverage estimates for bipolar disorder had poor agreement with, and were considerably lower than, estimates from the World Mental Health Surveys (3.1% *v.* 13.0% for low-income; 3.5% *v.* 13.0% for lower-middle-income; 3.1% *v.* 15.9% for upper-middle-income; 10.4% *v.* 28.4% for high-income; and 4.4% *v.* 22.9% overall). Differing methodologies likely explain this: the WMHS asks individuals whether they have received treatment from any mental health provider, whereas the Mental Health Atlas relies on health information systems reporting on mental health specialist service utilisation. However, the low coverage reported for bipolar disorder may also be partially attributed to frequent misdiagnoses of this condition (Singh and Rajput, 2006).

The depression service coverage estimates reported in this paper were very low. Given the understanding that mild cases of depression are rarely treated in specialist services, we also estimated service coverage for moderate-severe depression. These estimates were still considerably lower than those from the World Mental Health Surveys (4.3% *v.* 18.2% for lower-middle-income; 13.0% *v.* 31.1% for upper-middle-income; 31.1% *v.* 50.6% for high-income; and 10.7% *v.* 40.3% overall) (Thornicroft *et al*., 2017). This may be attributed to a lack of help-seeking behaviour of people with depression and may infer that the Mental Health Atlas' reporting method appears to be less reliable for depression. As with bipolar disorder, the WMHS asks individuals whether they have received treatment (compared with a reliance on health information systems), and the Mental Health Atlas only collects data from specialist mental health services. As shown in Atlas data, many countries continue to have highly centralised mental health systems often dominated by inpatient psychiatric hospitals which, for a variety of reasons, do not typically treat common mental disorders such as depression. As a result, people living with depression tend to seek help first from general physicians and other primary health care facilities (Bifftu *et al*., 2018) and are overlooked by the Mental Health Atlas method of data collection. Compounding issues around data availability is the general notion that mental health information systems are weak in outpatient services, the most appropriate facility for depression management.

There are several advantages to the method of estimating service coverage for severe mental disorders outlined in this study. For the first time, a method for assessing the validity of country-reported service utilisation data has been developed and tested; and the appropriateness of this method for estimating individual mental disorders has been explored. This method appears to be appropriate for psychosis; however, for mood disorders (including bipolar and depressive disorders), population-based surveys and data from non-specialist health facilities (such as primary healthcare centres) may be more effective methods.

The method of estimating service coverage outlined in this paper provides significant progress in tracking a critical mental health indicator. However, it has also highlighted that considerable work is still needed to develop global mental health information systems further. Several countries could not provide data for the service utilisation questions in the Mental Health Atlas 2017 questionnaire. For example, data availability on service coverage was remarkably low in the WHO regions of South East Asia and Africa. In these two regions, according to Atlas 2017, at least 20% of Member States reported that no data for mental health had been compiled during the last 2 years. The most common reason for non-reporting is that routine data collection is either absent or incomplete in these countries. For example, data may be only available at outpatient or inpatient facilities but not both; or data may be collected at the district or regional levels but not aggregated nationally.

Even when data are routinely collected, other challenges are observed – such as the absence of a unique patient identifier system which makes the process of identifying visits of each case or follow-up of cases after discharge a challenge leading to a risk of overcounting cases. Issues of correct reporting on disorder-specific data lie in the fact that the Atlas questionnaire does not request countries to report on the diagnostic system used in medical records and variations in the system; criteria and clinical expertise may vary within and between countries.

Some limitations in our approach to estimating global service coverage for mental disorders should be noted. The Mental Health Atlas 2017 data are restricted to service utilisation in specialist mental health services. Service utilisation data from primary care, private service providers, non-governmental organisations and other service providers are not captured. This is especially poignant for countries with few mental health services typically restricted to large cities. Data collection from sentinel primary care settings may be important to estimate service coverage for disorders such as depression.

Differences in case-definitions for psychosis were addressed through an adjustment of GBD 2016 prevalence estimates of schizophrenia (ICD-10: F20) to meet the Mental Health Atlas 2017 case-definition of psychosis (ICD-10: F20–29). We also adjusted GBD 2016 depression prevalence estimates to reflect the 12-month prevalence of moderate and severe cases only. The GBD 2016 definition of bipolar disorder includes cyclothymia (ICD-10: F31.0–F31.6, F31.8–F31.9, F34.0), whilst the Mental Health Atlas 2017 does not include cyclothymia but does include mania (ICD-10: F30–31). However, these differences are unlikely to significantly impact because the prevalence of cyclothymia is extremely low, and mania will typically be classified as bipolar I disorder (Akiskal *et al*., 2000). Furthermore, while GBD 2016 was able to control for case-definitions used in survey data stringently, the Mental Health Atlas 2017 is self-report by countries that typically rely on health information systems capturing clinical diagnosis, and there may be inconsistencies between case-definitions.

An additional difference in case-definitions exists within the derivation of service coverage thresholds described in the methods. While the WMHS reports service coverage estimates for psychosis, the Mental Health Atlas reports estimates for the narrower definition of non-affective psychosis. However, given our thresholds are very low, we do not anticipate a significant impact on our findings.

For the commitments by countries made for mental health in WHO's Mental Health Action Plan and UN's SDGs to be satisfactorily implemented, a robust monitoring system is essential. Service coverage for severe mental disorders is an essential indicator for this system. There is an urgent need to develop better methods to obtain information on this indicator and increase the capacities of countries to generate reliable data using a routine health information system. Any delay in doing this will compromise the achievement of stated objectives and targets of commitments countries have made.

It is important to note that the Mental Health Atlas, the tool used to measure coverage in this exercise, is an on-going activity of the WHO. As more accurate and comprehensive information on service coverage and other aspects of mental health systems become available, and the concepts and definitions of resources become more refined, it is expected that the database of the Mental Health Atlas will also become better organised and more reliable. While, in many cases, countries' information systems are weak, the Atlas exercise itself, including regular follow-up with countries every 2 years, may catalyse further development by demonstrating the utility of such systems.

## Supplementary material

For supplementary material accompanying this paper visit http://dx.doi.org/10.1017/gmh.2021.19. click here to view supplementary material
