# Global estimates of service coverage for severe mental disorders: findings from the WHO Mental Health Atlas 2017 – Addendum

**DOI:** 10.1017/gmh.2021.30

**Published:** 2021-08-03

**Authors:** Kara Jaeschke, Fahmy Hanna, Suhailah Ali, Neerja Chowdhary, Tarun Dua, Fiona Charlson

During original publication of Jaeschke, K., Hanna, F., Ali, S., Chowdhary, N., Dua, T., & Charlson, F. (2021). the disclaimer was not included. The disclaimer is as follows:

Disclaimer:

The authors alone are responsible for the views expressed in this Article and they do not necessarily represent the views, decisions, or policies of the institutions with which they are affiliated.
