# Supplementary material for: Global estimates of service coverage for severe mental disorders: findings from the WHO Mental Health Atlas 2017
Source: Glob Ment Health (Camb). 2021 Jul 21;8:e27. doi: 10.1017/gmh.2021.19 (PMC8320004; doi:10.1017/gmh.2021.19)
Supplement: Supplementary file 1 [file gmhsup.zip › S2054425121000194sup002.docx]

**Data Input:** Estimation of expected cases

Figure 1: Flow of data inputs, adjustments and outputs


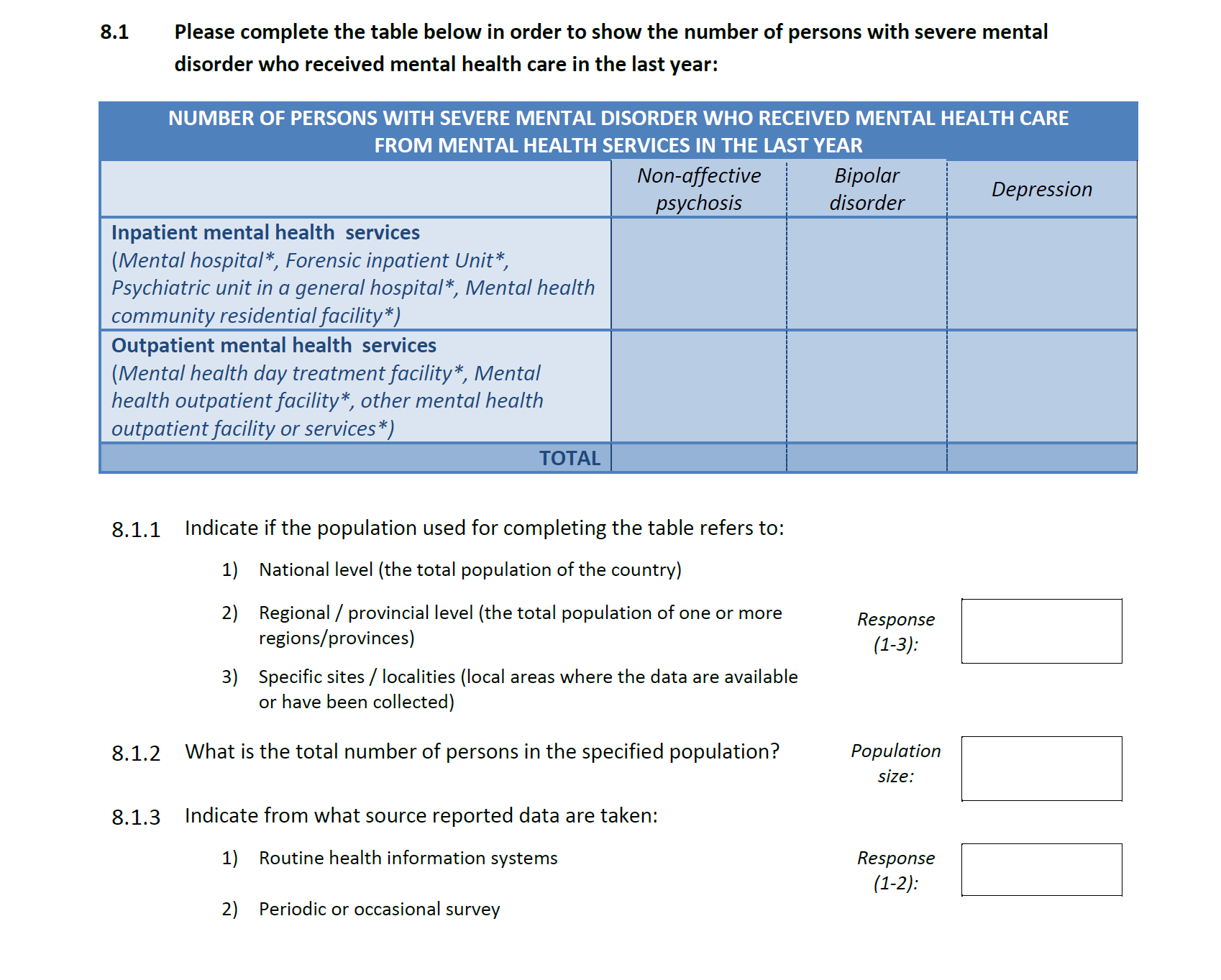


Figure S1: Section 8 of the Mental Health Atlas Questionnaire 2017


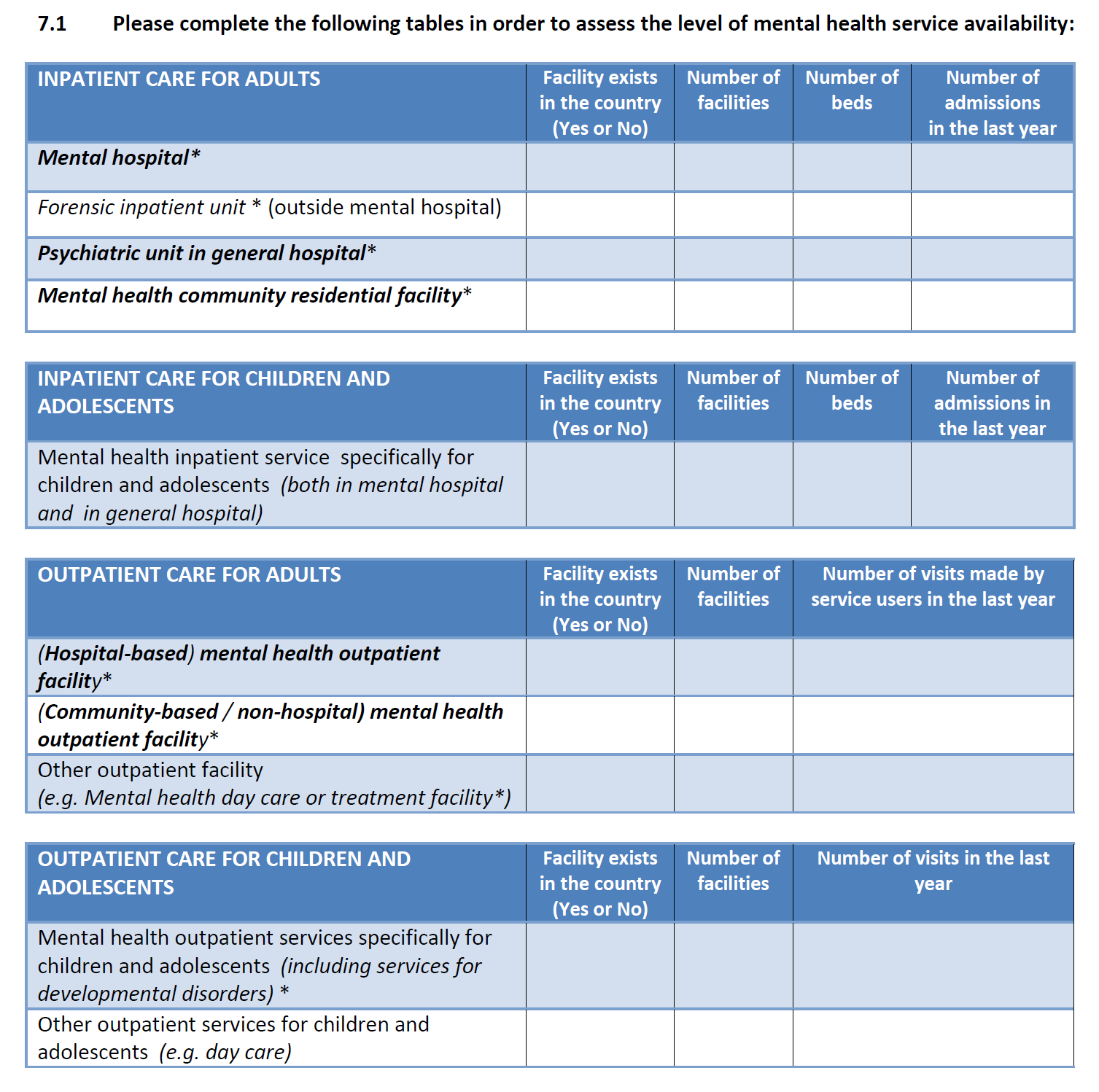

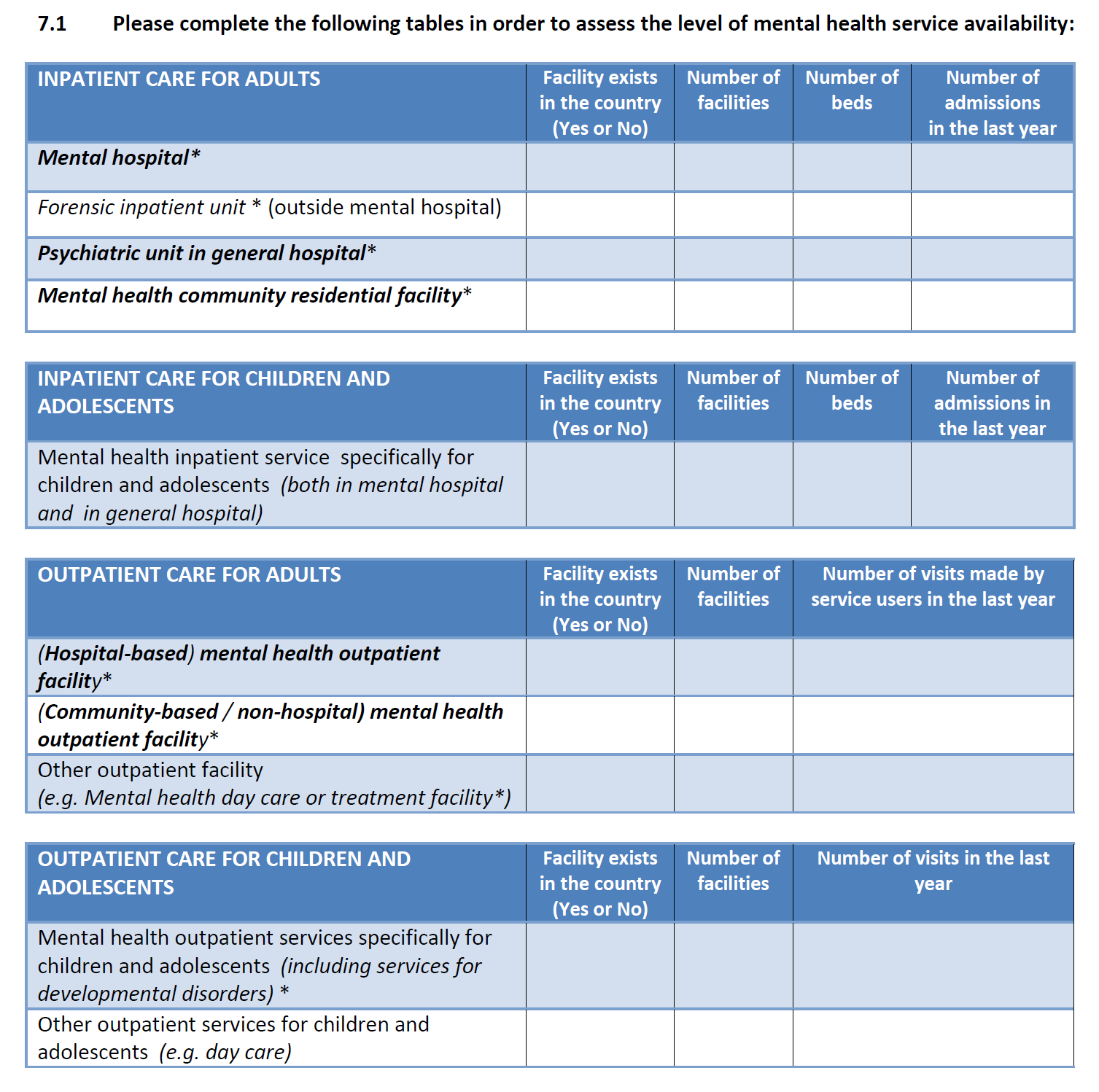


Figure S2: Section 7 of the Mental Health Atlas Questionnaire 2017

Table 1: Applied lower and upper service coverage estimate thresholds

| Income level | Country | Coverage | Lower CI | Upper CI |
| --- | --- | --- | --- | --- |
| Psychosis* | *** |  |  |  |
| Low income | Nigeria | 21.3% | 1.3% | - |
| Low-middle income | China | 11.0% | 0.4% | - |
| Upper-middle income | Lebanon | 20.1% | 9.9% | - |
| High-income | Japan | 24.2% | 14.4% | - |
| Depression* | **** |  |  |  |
| Low income | Nigeria | 21.3% | - | 41.3% |
| Low-middle income | Colombia | 27.8% | - | 37.2% |
| Upper-middle income | Mexico | 25.8% | - | 34.2% |
| High-income | Belgium | 60.9% | - | 78.7% |
| Bipolar disorder** | ***** |  |  |  |
| Low and low-middle income | All | 13.0% | - | 21.6% |
| Upper-middle income | All | 15.9% | - | 21.8% |
| High-income income | All | 28.4% | - | 32.3% |

*taken from ‘severe’ category of Table 3: 12-month service use by severity of mental disorders in the WMH surveys (29)

**taken from 12-month treatment from ‘any mental health service’ of Table 6: Lifetime and 12-month treatment of DSM-IV/CIDI Bipolar Spectrum Disorders (30).

*** The country listed has the lowest coverage in the WMH surveys for that income group. This is used as the lower threshold for psychosis coverage estimates.

****The country listed has the highest coverage in the WMH surveys for that income group. This is used as the highest threshold for depression coverage estimates.

*****For bipolar disorder, there are only global values from the WMH surveys, which are used as the highest threshold for bipolar coverage estimates. Note: missing values mean no thresholds were set.

Table 2: Estimates of service coverage for psychosis using Atlas 2017

| Grouping | n | Mean | Lower 95% CI | Upper 95% CI |
| --- | --- | --- | --- | --- |
| Income |  |  |  |  |
| Low income | 7 | 10.9% | 3.3% | 30.4% |
| Lower middle-income | 14 | 21.5% | 11.9% | 35.7% |
| Upper middle-income | 14 | 29.2% | 19.9% | 40.7% |
| High income | 15 | 59.5% | 42.9% | 74.1% |
| WHO region |  |  |  |  |
| African Region | 8 | 12.8% | 5.7% | 26.2% |
| Region of The Americas | 11 | 25.6% | 14.5% | 41.2% |
| Eastern Mediterranean Region | 6 | 16.1% | 3.4% | 51.4% |
| European Region | 16 | 56.8% | 44.3% | 68.4% |
| South-East Asia Region | 2 | 18.4% | 1.8% | 73.5% |
| Western Pacific Region | 7 | 34.7% | 20.2% | 52.8% |
| GBD region |  |  |  |  |
| High income | 11 | 51.5% | 35.0% | 67.6% |
| Latin America & Caribbean | 10 | 26.7% | 14.1% | 44.7% |
| Central Europe, Eastern Europe, and Central Asia | 9 | 56.5% | 37.4% | 73.9% |
| North Africa and Middle East | 5 | 24.3% | 5.9% | 62.2% |
| South Asia | 2 | 18.4% | 1.8% | 73.5% |
| Sub‐Saharan Africa | 9 | 10.0% | 4.1% | 22.3% |
| Southeast Asia, East Asia, and Oceania | 4 | 25.9% | 20.3% | 32.3% |
| Overall | **50** | **31.3%** | **22.9%** | **41.0%** |

Table 3: Estimates of service coverage for bipolar disorder using Atlas 2017

| Grouping | n | Mean | Lower 95% CI | Upper 95% CI |
| --- | --- | --- | --- | --- |
| Income |  |  |  |  |
| Low income | 7 | 3.1% | 0.8% | 11.5% |
| Lower middle-income | 15 | 3.5% | 1.8% | 6.8% |
| Upper middle-income | 20 | 3.1% | 1.8% | 5.5% |
| High income | 14 | 10.4% | 6.7% | 15.9% |
| WHO region |  |  |  |  |
| African Region | 9 | 2.6% | 0.9% | 7.2% |
| Region of The Americas | 14 | 4.2% | 2.6% | 6.7% |
| Eastern Mediterranean Region | 8 | 1.4% | 0.4% | 5.1% |
| European Region | 16 | 7.2% | 4.2% | 12.2% |
| South-East Asia Region | 1 | 21.7% | - | - |
| Western Pacific Region | 8 | 7.5% | 4.1% | 13.1% |
| GBD region |  |  |  |  |
| High income | 8 | 14.5% | 10.3% | 20.1% |
| Latin America & Caribbean | 13 | 4.1% | 2.4% | 6.7% |
| Central Europe, Eastern Europe, and Central Asia | 11 | 4.3% | 2.3% | 8.0% |
| North Africa and Middle East | 7 | 2.0% | 0.6% | 6.9% |
| South Asia | 7 | 2.0% | 0.6% | 6.9% |
| Sub‐Saharan Africa | 10 | 1.9% | 0.6% | 5.6% |
| Southeast Asia, East Asia, and Oceania | 7 | 8.5% | 4.3% | 16.0% |
| Overall | **56** | **4.4%** | **3.1%** | **6.3%** |

Table 4: Estimates of service coverage for moderate to severe depression using Atlas 2017

| Grouping | n | Mean | Lower 95% CI | Upper 95% CI |
| --- | --- | --- | --- | --- |
| Income |  |  |  |  |
| Low income | 8 | 2.9% | 1.3% | 6.3% |
| Lower middle-income | 16 | 4.3% | 2.3% | 7.9% |
| Upper middle-income | 22 | 13.0% | 8.0% | 20.3% |
| High income | 17 | 31.1% | 18.3% | 47.6% |
| WHO region |  |  |  |  |
| African Region | 10 | 3.4% | 2.2% | 5.3% |
| Region of The Americas | 14 | 14.6% | 8.2% | 24.6% |
| Eastern Mediterranean Region | 9 | 3.6% | 1.5% | 8.3% |
| European Region | 18 | 30.9% | 18.9% | 46.1% |
| South-East Asia Region | 3 | 11.0% | 1.0% | 61.1% |
| Western Pacific Region | 9 | 6.1% | 2.2% | 15.7% |
| GBD region |  |  |  |  |
| High income | 11 | 35.5% | 20.1% | 54.6% |
| Latin America & Caribbean | 13 | 15.0% | 8.1% | 26.2% |
| Central Europe, Eastern Europe, and Central Asia | 10 | 20.3% | 8.7% | 40.3% |
| North Africa and Middle East | 8 | 5.0% | 2.8% | 9.0% |
| South Asia | 2 | 3.7% | 0.6% | 19.4% |
| Sub‐Saharan Africa | 11 | 2.7% | 1.4% | 5.0% |
| Southeast Asia, East Asia, and Oceania | 8 | 6.9% | 2.2% | 19.8% |
| Overall | **63** | **10.7%** | **7.4%** | **15.2%** |

Table S1: Checklist of information that should be included in new reports of global health estimates

| Item # | Checklist item | Reported on page # |
| --- | --- | --- |
| Objectives and funding | | |
| 1 | Define the indicator(s), populations (including age, sex, and geographic entities), and time period(s) for which estimates were made. |  |
| 2 | List the funding sources for the work. |  |
| Data Inputs | | |
| *For all data inputs from multiple sources that are synthesized as part of the study:* | | |
| 3 | Describe how the data were identified and how the data were accessed. |  |
| 4 | Specify the inclusion and exclusion criteria. Identify all ad-hoc exclusions. |  |
| 5 | Provide information on all included data sources and their main characteristics. For each data source used, report reference information or contact name/institution, population represented, data collection method, year(s) of data collection, sex and age range, diagnostic criteria or measurement method, and sample size, as relevant. |  |
| 6 | Identify and describe any categories of input data that have potentially important biases (e.g., based on characteristics listed in item 5). |  |
| *For data inputs that contribute to the analysis but were not synthesized as part of the study:* | | |
| 7 | Describe and give sources for any other data inputs. |  |
| *For all data inputs:* | | |
| 8 | Provide all data inputs in a file format from which data can be efficiently extracted (e.g., a spreadsheet rather than a PDF), including all relevant meta-data listed in item 5. For any data inputs that cannot be shared because of ethical or legal reasons, such as third-party ownership, provide a contact name or the name of the institution that retains the right to the data. |  |
| Data analysis | | |
| 9 | Provide a conceptual overview of the data analysis method. A diagram may be helpful. |  |
| 10 | Provide a detailed description of all steps of the analysis, including mathematical formulae. This description should cover, as relevant, data cleaning, data pre-processing, data adjustments and weighting of data sources, and mathematical or statistical model(s). |  |
| 11 | Describe how candidate models were evaluated and how the final model(s) were selected. |  |
| 12 | Provide the results of an evaluation of model performance, if done, as well as the results of any relevant sensitivity analysis. |  |
| 13 | Describe methods for calculating uncertainty of the estimates. State which sources of uncertainty were, and were not, accounted for in the uncertainty analysis. |  |
| 14 | State how analytic or statistical source code used to generate estimates can be accessed. |  |
| Results and Discussion | | |
| 15 | Provide published estimates in a file format from which data can be efficiently extracted. |  |
| 16 | Report a quantitative measure of the uncertainty of the estimates (e.g. uncertainty intervals). |  |
| 17 | Interpret results in light of existing evidence. If updating a previous set of estimates, describe the reasons for changes in estimates. |  |
| 18 | Discuss limitations of the estimates. Include a discussion of any modelling assumptions or data limitations that affect interpretation of the estimates. |  |
