# Supplementary figures and images for: Global estimates of service coverage for severe mental disorders: findings from the WHO Mental Health Atlas 2017
Source: Glob Ment Health (Camb). 2021 Jul 21;8:e27. doi: 10.1017/gmh.2021.19 (PMC8320004; doi:10.1017/gmh.2021.19)

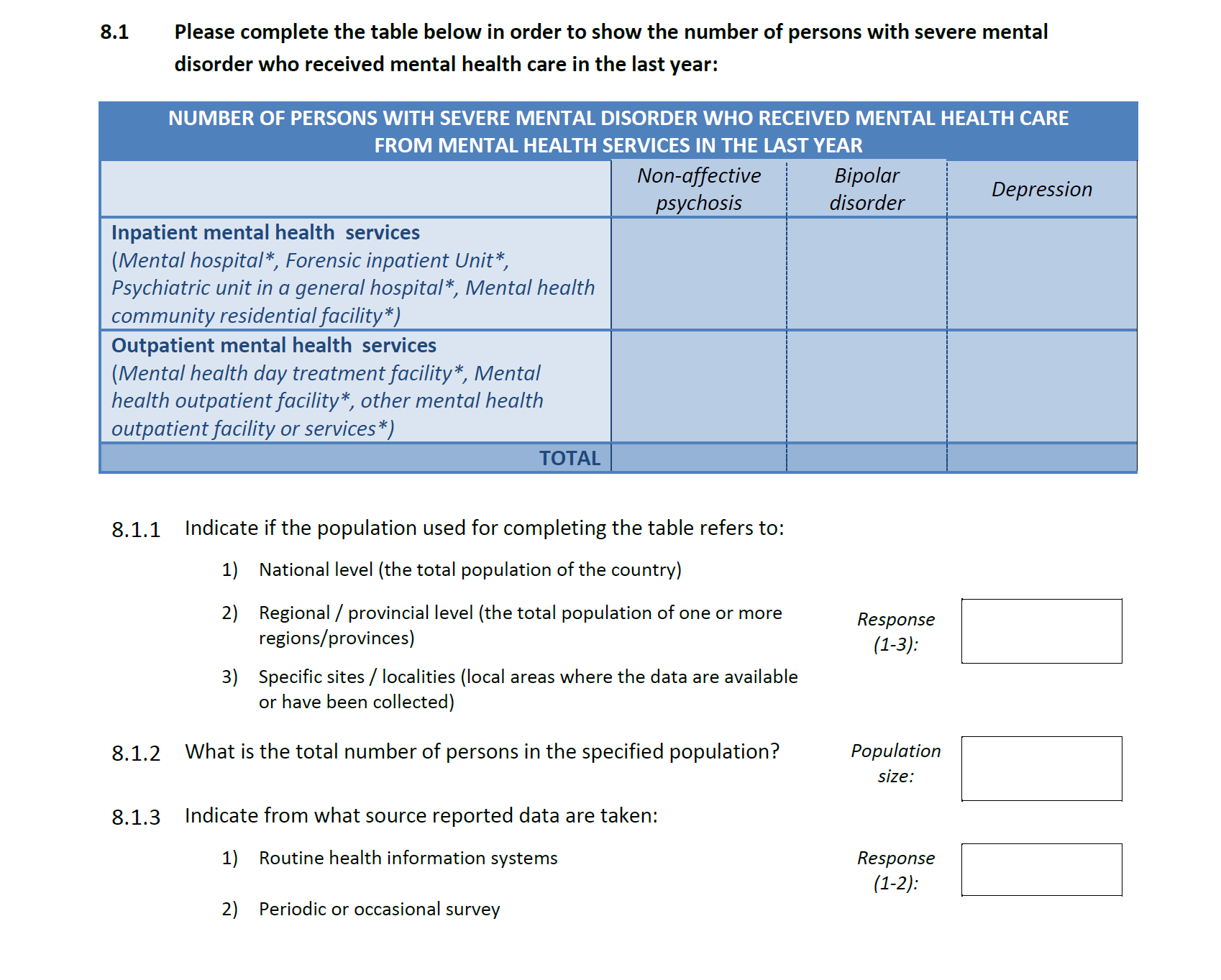


Figure S1: Section 8 of the Mental Health Atlas Questionnaire 2017

Supplement: Supplementary file 1 [file gmhsup.zip › S2054425121000194sup001.docx]
